# The National Medical Union (Official)

**Published:** 1914-03-07

**Authors:** 


### THE NATIONAL MEDICAL UNION (Official).

The second annual dinner of the Lewisham and District Non-Panel Doctors took place at Lewisham on Tuesday evening, February 24. The thirty gentlemen present represented the Lewisham Branch of the London Medical Committee and the Deptford and District Non-Panel Association, both of which are federated with the National Medical Union. The local branch was instituted in January 1913, and the first dinner was held last year at the Ship Hotel, Greenwich.

The Chairman, Dr. C. H. Hartt, of Greenwich, in giving the toast of the evening, "The Non-Panel Doctors of S.E. London, including the Boroughs of Lewisham, Greenwich, Deptford, and Camberwell, and Success to the National Medical Union," said: "After all it is a source of gratification to you to know that, after twelve months of panel in our midst, we have survived the ordeal and in goodly numbers are able to meet and discuss, not only the past, but the future. This gathering to-night speaks well for the future and shows that we are not downhearted. It is probable that we are not as well off financially as if we had gone on the panel, but at least we have the satisfaction of knowing that we have not broken our obligation and word of honour by joining a system that, in my opinion, is a fraud upon insured persons. To-day we meet strengthened in the belief that we acted wisely in refusing to give the panel system any assistance, and resolved that in future it will be impossible for any Government to legislate without consultation with those who understand the wants and requirements of general practitioners, and in a way that is just and fair to insured persons.

"Twelve months ago we were caught napping; we were unprepared. We will take care that it does not occur again. Nations and peoples have discovered, when it is too late, that want of preparedness has been, and is, the cause of much suffering and even ruin. The National Medical Union that is now so well started will fulfil a great want in the ranks of our profession. It aims at combination and will be the means by which we shall regain our lost position. Had our Union existed a year ago, and had Mr. Lloyd George known of such a combination, he would not have gone for advice to quasi-politicians of the type of Drs. Addison, Victor Horsley, and Lauriston Shaw, but would have probably sent for and taken the advice of practitioners like ourselves. Instead, he used all the meanest devices possible in inflicting upon us a thraldom that no honourable man could accept. After all, we ask for nothing but what is right—fair play.

"I have said that the National Insurance Act is a fraud. Let me justify that statement. Take Section 15, Clause 3, the Own Arrangement Clause. Mr. Lloyd George stated at the Tabernacle that every insured person was to have the right to select his own doctor, free choice. Has Section 15, Clause 3, been carried out according to the manner in which the members of the House of Commons intended? No.

"The Act is a failure. Men of the type of Sir Charles Macara, that great employer of labour in the North of England, have described it as unworkable, and even Sir John Collie condemns it and takes upon himself to propose in its place a State Medical Service. In conclusion, let us ask ourselves why the Act is such a failure. In the first place it was not properly thought out, and, secondly, it takes away the freedom of individuals. What is life without freedom of thought and action? It makes life intolerable. Then let us, as freemen, as professional men, combine so as to resist undue or unnecessary interference on the part of the State. Better to be free men and poor than slaves of wealth."

Dr. Macnamara, in replying for the Union, said that those of them who had gone through the trials and struggles of the past few years—many of them suffering financial loss, yet preserving throughout it all their personal, professional, and social honour—knew what a hard task it was to speak calmly of their colleagues who had participated with them in that fight of January 13. It might be possible to look with a kindly eye on those miserable weaklings who—to adopt from Mr. Lloyd George's repertoire of English undefiled—fled like shorn lambs before the biting blast or drifting sleet. But it required charity, more than superhuman, to think of the men who associated themselves with him in bringing them to the morass in which their profession was now engulfed.

They were now in the position of trying to formulate some ground-work, from which they might restore the profession to the position of honour it previously held. As opponents to the Act they set out to secure six cardinal points. Free choice of doctor was one of them. That they had not secured; in fact the only asset in the whole business, on which any members of the profession could congratulate themselves, was that they had approached the adequate remuneration they asked for. With that he supposed the panel men were satisfied, and if so, it was no business of theirs. Now, he urged, it was necessary for all who believed in the independence and freedom of the profession to join the ranks of the National Medical Union. It would be necessary, for when 1915 came an attempt would be made to reduce the present capitation fee. The doctors working the Act were getting too much. The *Daily News* said so, and it ought to know. (Laughter.) In his concluding remarks he insisted that officialism could never replace the spirit of humanity. He was hoping, perhaps against hope, that the panel men might at last see the error of their way.

Dr. Halliwell related that he had met a practitioner with between four and five thousand names on his panel, who said he attended between 160 and 180 a day. He (the speaker) said to him, "You cannot possibly examine them." He said, "I see them all right enough and give them a prescription." He (the speaker) replied, "Well, you cannot examine their chests and see if there is anything the matter, and use the stethoscope." He said, "Well, I have not seen that for a fortnight."

Dr. J. Clarke, of Woolwich, said that he personally was unique in the fact that he had been in the wilderness and sown his wild oats and had come back. He had been on the panel. His reasons for going on were known to many of them. He went on the panel for the sole purpose of keeping his colleagues together. What were his reasons for coming off? His chief reason was, as he had put it to scores of his patients, "I felt that under this Act I could not do you the amount of good that I could free." He was glad to say that his patients had largely believed him, and that brought him back to the question of officialism and humanity. In giving up the panel work he gave up £500 a year, because he had always had a large practice. They might ask if he had been better off since. He could assure them that he had been better off in health. Under the panel system they lived in an atmosphere of worry, and their health was suffering as a result. Mr. Jones or Mr. Smith might pick a little quarrel over the most trivial things, and for ever it seemed as if there were held over their heads the sword of Damocles.

"A young man of twenty-six, a panel patient," Dr. Clarke related in giving some of his experiences, "walked into my surgery one Saturday evening, when I had thirty patients waiting, and actually asked me to cut his corns. Needless to say I told him to retire in very lurid language." (Laughter.)

Dr. F. S. Barnett, in submitting "The Chairman," remarked that they were under a deep sense of gratitude to him for coming in spite of the trouble he had in his domestic life in the illness of his wife. He attended that night at Mrs. Hartt's own wish. She was keenly interested, and wished him to be there. Some speakers had said that they, as non-panel medical men, were financially at a discount over this Act. Were they? He did not think they were. Although they might have lost something financially in not joining the panel, he was sure that they were having an easier time, were paid better, and were getting their patients free to themselves. Even their old patients who had paid into the Insurance Fund were glad to come back and be treated by them.

The toast was received with musical honours, the Chairman briefly replying.

---

### Constitution Committee.

A meeting of the Constitution Committee was held at 346 Strand, on Friday, February 27. Dr. O'Connor, who was present, maintained the view that, until a clear statement of the objects of the Union has been laid down, it was premature to proceed to the discussion of a Constitution, and, after an informal discussion upon some of the points mooted, the meeting adjourned.

### MANCHESTER DISTRICT.

### Meeting of Executive Committee.

The Executive Committee of the Manchester District met on Thursday, February 26, under the chairmanship of W. Coates, Esq., C.B. The following members were also present:—Professor G. A. Wright, Drs. Brassey Brierley, Pinder, Wheeler-Hart, Stenhouse, A. C. Clarke, Grange, Malim, C. E. M. Lowe, E. W. Floyd, McMillan, Stocks, and Prowse.

It was resolved that an organisation sub-committee be appointed (with powers of co-option) to deal with the enrolment of members, and to report progress at each meeting of the Executive Committee.

Dr. Pinder read the preliminary statement of some evils consequent on the administration of the Medical Benefit Section of the National Insurance Act, which had been drawn up by the sub-committee appointed at the last meeting. It was decided that the statement should be sent to the lay press as soon as convenient as a first instalment of an indictment of the Acts, and that the sub-committee should continue the work of collecting and recording evidence for future publication.

Dr. C. E. M. Lowe (Crewe) introduced a resolution outlining a scheme of policy for the National Medical Union under five heads:—

1. To strive for complete freedom of doctor and patient.
2. To aid non-panel practitioners in every way possible in their present unequal struggle.
3. To explain to the public the dangers to national health, and the injustices to insured people and to the profession, which arise from the administration of the Acts.
4. To lay down the principles which should guide the framing of amendments to the Acts.
5. To organise a scheme of personal canvass for the purpose of inducing non-panel men to join the Union and help in these objects.

It was resolved that the amendment sub-committee be empowered to draft a resolution embodying the scheme outlined by Dr. Lowe for transmission to the Central Council.

Dr. W. P. Stocks was nominated as member of the Central Federating Council to represent the Southern Division of the Manchester District.

---

### THE INSURANCE ACT.

### Sanatorium Deficiencies.

The deficiencies in sanatorium accommodation in many areas have already been noted several times in The Hospital, and a letter about the position in one county appears in our correspondence columns this week. Salford has especially distinguished itself by the failure of its Insurance Committee to provide sanatorium accommodation; and we are glad to note that some of the members of the latter are beginning to realise the facts of the case, and to press for the fulfilment of the Committee's obligations.

